# Synergistic Apoptosis-Inducing Antileukemic Effects of Arsenic Trioxide and *Mucuna macrocarpa* Stem Extract in Human Leukemic Cells via a Reactive Oxygen Species-Dependent Mechanism

**DOI:** 10.1155/2012/921430

**Published:** 2011-07-27

**Authors:** Kuan-Hung Lu, Hui-Ju Lee, Min-Li Huang, Shang-Chih Lai, Yu-Ling Ho, Yuan-Shiun Chang, Chin-Wen Chi

**Affiliations:** ^1^Graduate Institute of Chinese Pharmaceutical Sciences, China Medical University, Taichung 404, Taiwan; ^2^Institute of Biomedical Sciences, Academia Sinica, Taipei 115, Taiwan; ^3^Center of General Education, National Taipei University of Nursing and Health Sciences, Taipei 112, Taiwan; ^4^Department of Health and Nutrition Biotechnology, Asia University, Taichung 413, Taiwan; ^5^Department of Nursing, Hungkuang University, Taichung 433, Taiwan; ^6^Department of Medical Research and Education, Taipei Veterans General Hospital, Taipei 112, Taiwan; ^7^Department and Institute of Pharmacology, School of Medicine, National Yang-Ming University, Taipei 112, Taiwan

## Abstract

The objective of this study was to examine the potential of enhancing the antileukemic activity of arsenic trioxide (ATO) by combining it with a folk remedy, crude methanolic extract of *Mucuna macrocarpa* (CMEMM). Human leukemia cells HL-60, Jurkat, and Molt-3 were treated with various doses of ATO, CMEMM, and combinations thereof for 24 and 48 h. Results indicated that the combination of 2.5 **μ**M ATO and 50 **μ**g/mL CMEMM synergistically inhibited cell proliferation in HL-60 and Jurkat cell lines. Apoptosis triggered by ATO/CMEMM treatment was confirmed by accumulation of cells in the sub-G_1_ phase in cell cycle analyses, characteristic apoptotic nuclear fragmentation, and increased percentage of annexin V-positive apoptotic cells. Such combination treatments also led to elevation of reactive oxygen species (ROS). The antioxidants *N*-acetyl cysteine (NAC), butylated hydroxytoluene, and **α**-tocopherol prevented cells from ATO/CMEMM-induced apoptosis. The ATO/CMEMM-induced activation of caspase-3 and caspase-9 can be blocked by NAC. In summary, these results suggest that ATO/CMEMM combination treatment exerts synergistic apoptosis-inducing effects in human leukemic cells through a ROS-dependent mechanism and may provide a promising antileukemic approach in the future.

## 1. Introduction

Arsenic is one of the oldest drugs in both Western and traditional Chinese (TCM) medicines. More than 2,000 years ago, it was first used to treat various diseases from syphilis to cancer [1]. Because it is both a therapeutic agent and a poison, arsenic was used only to treat severe diseases with the principle of “taming an evil with a toxic agent” in TCM [2]. In recent years, the clinical efficacy of arsenic trioxide (ATO) has been well characterized in the treatment of newly diagnosed and relapsed acute promyelocytic leukemia (APL) [3]. The combination of ATO and all-*trans* retinoic acid is a very effective new strategy for APL patients who are unable to tolerate conventional therapy [4]. In addition, in vitro studies showed that ATO apparently affected numerous intracellular signal transduction pathways to alter cellular functions. Therapeutic benefits of ATO treatment involve antiproliferation, antiangiogenesis, promotion of differentiation, and induction of apoptosis in a wide variety of malignancies, including both hematologic cancer and solid tumors [5, 6]. However, clinical trials indicate that ATO as a single agent has not demonstrated significant benefit in a variety of non-APL hematological malignancies [7–9].

*Mucuna macrocarpa* Wallich (Leguminosae) is a large woody climber distributed throughout Taiwan and Southeastern Asia. In folk medicine, dried stems of this plant have been used to fortify blood circulation for various hematologic and circulatory related ailments [10]. A previous study has reported that crude methanolic extract of *M. macrocarpa* (CMEMM) exerts antileukemic effects by induction of apoptosis in HL-60 human leukemia cells in vitro and in vivo, providing thus scientific support of its putative clinical efficacy [11]. In addition, our preliminary study showed that the CMEMM-induced apoptosis in human leukemia cells might result from elevation of intracellular reactive oxygen species (ROS). Since generation of a moderate prooxidant environment may offer potential therapeutic opportunities in human leukemia cells, it will be a good strategy to enhance ATO cytotoxicity by adding a prooxidant agent [12]. Moreover, recent reports have demonstrated that ATO combined with natural components such as dietary isothiocyanates or flavonoids could improve the efficiency of ATO as an antileukemic drug [13–15].

For these reasons, we investigated the capacity of CMEMM to enhance ATO cytotoxicity in human leukemia cells (HL-60, Jurkat, and Molt-3). In the present study, antiproliferative activity was evaluated by the trypan blue exclusion assay. Apoptosis induced by combination of ATO and/or CMEMM was determined by typical apoptotic morphologic changes, cell cycle analyses, and Western blotting. Also, changes in ROS production and glutathione (GSH) levels were assessed. Furthermore, the free radical scavenger *N*-acetyl cysteine (NAC), butylated hydroxytoluene (BHT), *α*-tocopherol, and caspase inhibitor z-VAD-fmk were employed to further define the apoptotic mechanisms of the ATO/CMEMM treatment.

## 2. Materials and Methods

### 2.1. Reagents

RPMI-1640 medium, fetal bovine serum (FBS), gentamicin, trypan blue dye solution, and phosphate-buffered saline (PBS; pH 7.4) were purchased from Gibco (Grand Island, NY, USA). Arsenic trioxide, *N*-acetyl cysteine, BHT, *α*-tocopherol, dimethyl sulfoxide (DMSO), dihydroethidium (DHE), and 4′,6-diamidino-2-phenylindole (DAPI) were purchased from Sigma (St. Louis, MO, USA). Methanol was purchased from Merck (Darmstadt, Germany). Cycle TEST PLUS DNA Reagent Kit was purchased from Becton Dickinson (San Jose, CA, USA). ANNEX100F Kit (annexin V: FITC assay kit) was purchased from AbD Serotec (Kidlington, UK). M-PER mammalian protein extraction reagent was purchased from PIERCE (Rockford, IL, USA). Protease inhibitors cocktail and z-VAD-fmk were purchased from Calbiochem (Darmstadt, Germany). Mouse monoclonal antibody against caspase-3 was purchased from Imgenex (San Diego, CA, USA). Rabbit polyclonal antibodies against caspase-9, *β*-actin, and poly-ADP ribose polymerase (PARP) were purchased from Imgenex, Sigma and Cell Signaling Technology (Danvers, MA, USA), respectively. Enhanced chemiluminescent (ECL) detection reagent was purchased from PerkinElmer (Waltham, MA, USA).

### 2.2. Plant Material

*Mucuna macrocarpa* Wallich (Leguminosae) was collected in Nantou County, Taiwan. The material was identified by Professor Yuan-Shiun Chang of the Graduate Institute of Chinese Pharmaceutical Sciences, China Medical University. Crude methanolic extract of the stems of *M. macrocarpa *(CMEMM) was prepared by extraction with methanol and standardized by high-performance liquid chromatography (HPLC) fingerprint as described in detail in the preceding works [11].

### 2.3. Cell Culture and Drug Treatment

Human leukemia cell lines HL-60 (acute promyelocytic leukemia), Jurkat, and Molt-3 (acute T-lymphoblastic leukemia) were obtained from the American Type Culture Collection (ATCC, Rockville, Md). Cells were cultured in RPMI-1640 medium containing 10% FBS and 0.01 mg/mL gentamicin and incubated in a humidified atmosphere of 5% CO_2_ at 37°C. ATO and CMEMM were dissolved in 0.1 N NaOH and DMSO, respectively, and diluted in culture medium. The final concentrations of NaOH and DMSO were not more than 5 × 10^−5^ N and 0.1%, which did not affect cell viability. Cells were treated with 0, 2.5 and 5 *μ*M of ATO in combination with 0, 25, 50, and 75 *μ*g/mL of CMEMM for 24 and 48 h and then harvested for various analyses.

### 2.4. Cell Proliferation Assay

Cell growth was determined by the trypan blue exclusion assay. Cells were collected at scheduled times following CMEMM and/or ATO exposure. After centrifugation, cells were resuspended in culture media and stained with 0.4% trypan blue solution. Viable cells were counted using a hemocytometer. The percentages of cell growth were calculated by comparing the cells number with that of the control.

### 2.5. Cell Cycle Analysis

DNA staining was carried out using Cycle TEST PLUS DNA Reagent Kit. In brief, cells (1 × 10^6^ cells/mL) were washed and stained for DNA content according to the kit protocol. Fluorescence intensity of propidium iodide (PI) was determined using a FACScan flow cytometer and analyzed by ModFit LT software (Verity Software House, Topsham, ME, USA).

### 2.6. Cytological Examination

DAPI nucleic acid stain was used to observe apoptotic morphology of individual cells. Briefly, cells (1 × 10^6^ cells/mL) were washed once with PBS, collected on microscope slides by cytospin (Shandon Cytospin Cytocentrifuge), fixed with 10% neutral buffered formalin, and stained with 2.5 *μ*g/mL DAPI solution at room temperature. Photographs of the slides were taken under an inverted fluorescence microscopy (Olympus AX-10). Fragmented nuclei were suggestive of apoptosis.

### 2.7. ROS Production Measurement

The probe DHE was used for detection of cytosolic superoxide anion (O^2−^). Briefly, cells (1 × 10^6^ cells/mL) were washed once with PBS and stained with DHE-containing RPMI-1640 medium (without phenol red) at a final concentration of 10 *μ*M. After 30 minutes of incubation in a water bath at 37°C, samples were placed on ice for 10 minutes. Then, intracellular ROS levels were immediately examined using a FACScan flow cytometer and analyzed by CellQuest software (Becton Dickinson, San Jose, CA, USA). Data are expressed as mean fluorescence of ethidium.

### 2.8. Annexin V-FITC/PI Analysis

Annexin V-FITC/PI staining method was used to identify and quantify apoptotic cells. Cells (2 × 10^5^) were treated according to the manufacturer's instruction of ANNEX100F Kit. FITC/PI fluorescence intensity was measured by flow cytometry to differentiate between viable (annexin V-negative and PI-negative), early apoptotic (annexin V-positive, PI-negative), and late apoptotic (annexin V-positive and PI-positive) cells. The extent of apoptosis was quantified as percentage of annexin V-positive cells.

### 2.9. Western Blotting Analysis

To obtain total cellular protein extracts, cells (2 × 10^6^) were washed once with PBS and cell lysates were prepared by M-PER mammalian protein extraction reagent supplemented with protease inhibitors cocktail following the manufacturer's protocol. Cell lysates containing equal protein amounts were analyzed by Western blotting as previously described [16]. In brief, samples were subjected to electrophoretic separation in 12% SDS-polyacrylamide gels, blotted onto nitrocellulose membranes, and probed with primary antibodies against PARP, caspase-3, caspase-9, and *β*-actin. Following wash cycles with TBS-T buffer (Tris-Buffer Saline with Tween 20), membranes were incubated with horseradish peroxidase-conjugated secondary antibodies. Immunoreactive bands were visualized using the ECL detection reagent and Kodak medical X-ray processor.

### 2.10. Drug Combination and Statistical Analysis

To assess the interaction of two drugs such as synergism, additivity, or antagonism, the data of cell proliferation assay were analyzed by CalcuSyn V2 for Windows software (Biosoft, Cambridge, UK). The combination indices (CIs) were calculated based on the multiple drug effect equation of Chou and Talalay, where CI < 1, = 1, and >1 indicate synergism, additive effect, and antagonism, respectively [17, 18]. Each CI value represents the mean of three independent experiments which were performed in duplicates. Statistical significant differences between the control and ATO/CMEMM-treated groups were estimated by one-way analysis of variance (ANOVA) followed by Student-Newman-Keuls test using the SPSS for Windows 10.0 version software. Differences were defined as significant when *P* < 0.05.

## 3. Results

### 3.1. ATO and CMEMM Synergistically Inhibit Cell Growth on HL-60 Cells

**Figure 1:**
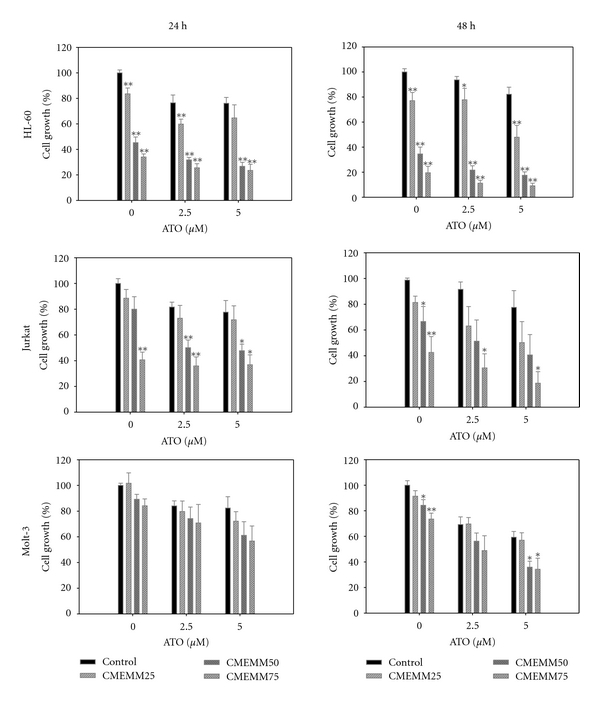
Antiproliferative effects of combined application of arsenic trioxide (ATO) and crude methanolic extract of *Mucuna macrocarpa* (CMEMM) on human leukemia cells. HL-60, Jurkat, or Molt-3 cells (1 × 10^5^ cells/mL) were seeded into 6-well plates and exposed to 0, 2.5, or 5 *μ*M ATO alone or together with 0, 25, 50, or 75 *μ*g/mL CMEMM for 24 and 48 h. Vehicle control cells were treated with 0.1% DMSO in medium. The percentages of cell growth were measured by the trypan blue exclusion assay and calculated by comparing the cells number with that of the vehicle controls. Each value represents the mean ± S.E. of duplicate cultures from three independent experiments. *P < 0.05, **P < 0.01 indicate significant difference from the respective control value.

To evaluate whether ATO and CMEMM present additive, synergistic, or antagonistic effect, HL-60, Jurkat, and Molt-3 cells were treated with 2.5 or 5 *μ*M ATO alone or in combination with increasing doses of CMEMM from 25 to 75 *μ*g/mL. As shown in Figure 1, CMEMM was found to enhance HL-60, Jurkat, and Molt-3 cells to the growth inhibitory effects of ATO for 24 or 48 h treatment. CI values for the combination of ATO and CMEMM after 24 h of treatment were calculated and shown in Table 1. Combination treatment of ATO and CMEMM synergistically inhibited cell growth in HL-60 cells. However, when combining 2.5 *μ*M ATO with 75 *μ*g/mL CMEMM or 5 *μ*M ATO with 25 *μ*g/mL CMEMM, the drug interaction showed antagonism in Jurkat or Molt-3 cells, respectively.

### 3.2. ATO/CMEMM Combination Triggers Apoptosis in Leukemic Cells

**Figure 2:**
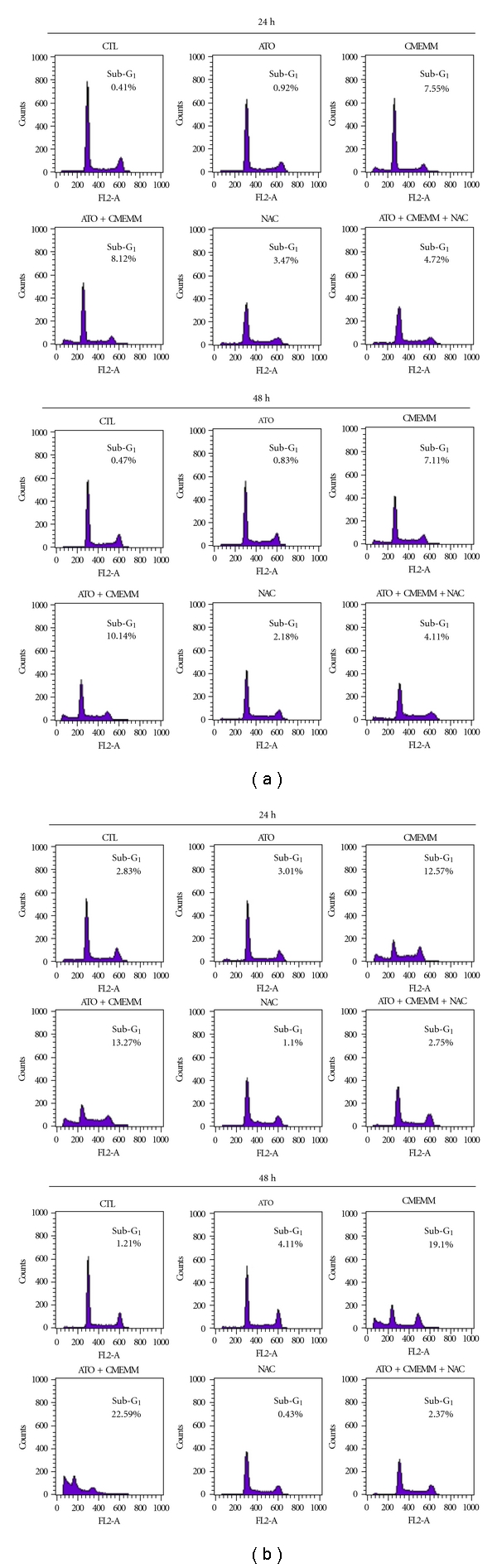
Cell cycle progression in leukemia cells exposed to arsenic trioxide (ATO) and/or crude methanolic extract of *Mucuna macrocarpa* (CMEMM). HL-60 (a) or Jurkat (b) cells (1 × 10^5^ cells/mL) were first treated with 5 mM *N*-acetyl cysteine (NAC) or untreated, followed by treatment with 0.1% DMSO (CTL), 2.5 *μ*M ATO, and/or 50 *μ*g/mL CMEMM as indicated. After 24 or 48 h of treatment, cells were collected and stained with propidium iodide and determined for DNA content using flow cytometry. The percentages of sub-G_1_ or hypodiploid cells were analyzed by ModFit LT software. The representative cell cycle progressions in ATO- and/or CMEMM-treated or control cells were from one of three independent experiments.

**Figure 3:**
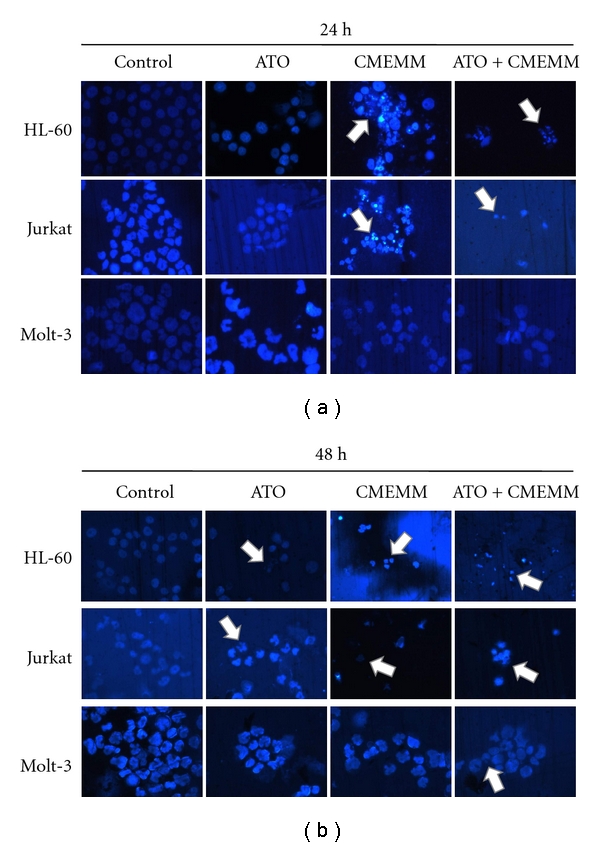
Nuclear morphological changes induced by arsenic trioxide (ATO) and/or crude methanolic extract of *Mucuna macrocarpa* (CMEMM). HL-60, Jurkat, or Molt-3 cells (1 × 10^5^ cells/mL) were treated with 0.1% DMSO (control), 2.5 *μ*M ATO, 50 *μ*g/mL CMEMM, or 2.5 *μ*M ATO plus 50 *μ*g/mL CMEMM. After 24 or 48 h of incubation, cells were washed with PBS and collected on microscope slides by cytospin. The nuclei were stained with 2.5 *μ*g/mL DAPI. Arrows indicate apoptotic bodies of nuclear fragmentation. Magnification × 200.

Since combination treatment of ATO and CMEMM resulted in reduced cell growth, cell cycles of HL-60 and Jurkat cells were analyzed using flow cytometry. As shown in Figure 2, treatments with ATO alone had negligible or very low apoptosis in leukemic cells, as measured by the accumulation of cells in the sub-G_1_ phase (or hypodiploid apoptotic cells). However, cotreatment of 2.5 *μ*M ATO and 50 *μ*g/mL CMEMM for 24 h increased cells in the sub-G_1_ phase. As treatments prolonged up to 48 h, the accumulation of cells in the sub-G_1_ phase increased more obviously in Jurkat cells (Figure 2(b)) than in HL-60 cells (Figure 2(a)). Additionally, as shown in Figure 3 of nuclear morphological changes in 2.5 *μ*M ATO- and/or 50 *μ*g/mL CMEMM-treated cells, ATO/CMEMM-treated cells presented with fragmented chromatin and formation of apoptotic bodies, which were in clear contrast with the intact nuclei of control groups. The apoptotic morphology results together with the flow cytometry data suggest that cotreatment exerted higher apoptosis-inducing effect than treatment of ATO or CMEMM alone.

### 3.3. ATO/CMEMM Combination Induces Apoptosis in Leukemic Cells through a ROS-Dependent Mechanism

**Figure 4:**
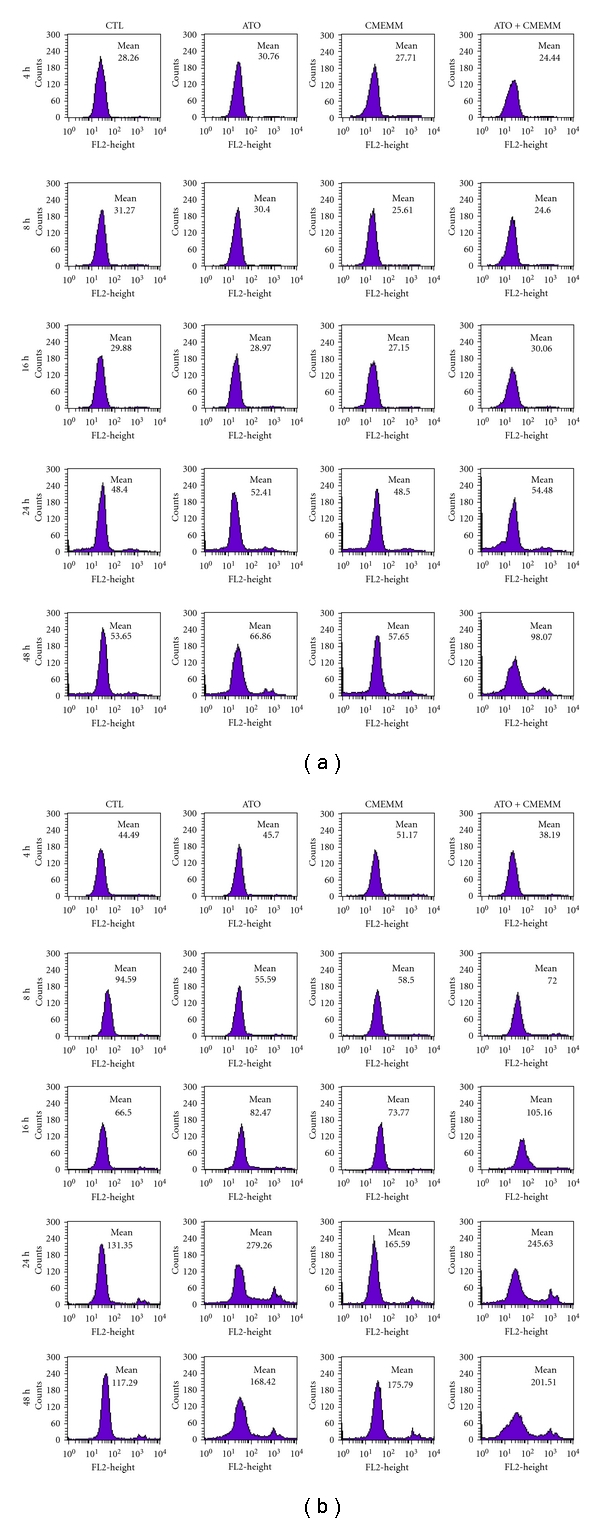
Changes in the level of intracellular reactive oxygen species (ROS) in leukemia cells exposed to arsenic trioxide (ATO) and/or crude methanolic extract of *Mucuna macrocarpa* (CMEMM). HL-60 (a) or Jurkat (b) cells (1 × 10^5^ cells/mL) were treated with 0.1% DMSO (control), 2.5 *μ*M ATO, 50 *μ*g/mL CMEMM, or 2.5 *μ*M ATO plus 50 *μ*g/mL CMEMM for 4 to 48 h. Then, cells were washed with PBS, incubated with dihydroethidium for 30 min, and analyzed for red fluorescence by flow cytometry. The mean fluorescence intensity was used as read-out for intracellular ROS levels.

**Figure 5:**
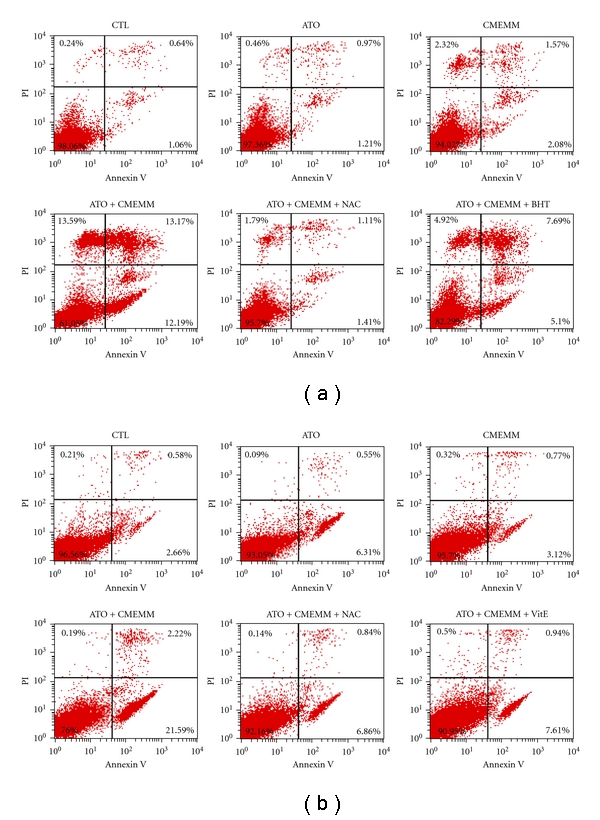
Annexin V-FITC/propidium iodide (PI) analyses of arsenic trioxide (ATO)- and/or crude methanolic extract of *Mucuna macrocarpa* (CMEMM)-treated cells. HL-60 (a) and Jurkat (b) cells (1 × 10^5^ cells/mL) were first treated with 5 mM *N*-acetyl cysteine (NAC), 50 *μ*M butylated hydroxytoluene (BHT), or 40 *μ*M *α*-tocopherol (VitE) or untreated, followed by treatment with 0.1% DMSO (CTL), 2.5 *μ*M ATO, and/or 50 *μ*g/mL CMEMM as indicated for 24 h. Quantitative percentages of apoptotic cells of ATO/CMEMM-treated cells were measured by flow cytometry. Data represent the result from one of three independent experiments.

Since ATO or other DNA damaging agents may trigger off the apoptotic pathway through the production of ROS, we sought to assess the role of ROS generation in ATO/CMEMM-induced apoptosis using the oxidant sensitive probe DHE. As shown in Figure 4, intracellular ROS content of ATO/CMEMM-treated HL-60 and Jurkat cells increased in a time-dependant manner within 16 to 48 h and 4 to 48 h periods, respectively. To further demonstrate that the apoptosis triggered by ATO-CMEMM treatment is mediated by higher production of ROS, the effect of the free radical scavenger NAC was evaluated. The possibility of direct interaction between ATO and NAC was already excluded in a previous report [19]. As shown in Figure 2, addition of 5 mM NAC treatment decreased the accumulation of cells in sub-G_1_ phase as compared to those observed in ATO/CMEMM-treated cells. Furthermore, the annexin V-FITC/PI assay was employed to investigate whether other antioxidant agents could also attenuate the apoptosis induced by cotreatment of ATO and CMEMM in HL-60 and Jurkat cells. As shown in Figure 5, the percentage of apoptotic cells (annexin V-positive) increased from 2.18% (ATO only) or 3.65% (CMEMM only) to 25.36% (ATO + CMEMM) in ATO/CMEMM-treated HL-60 cells after 24 h of exposure. Using the same treatment, the percentage of apoptotic cells increased from 6.86% (ATO only) or 3.89% (CMEMM only) to 23.81% (ATO + CMEMM) in ATO/CMEMM-treated Jurkat cells. The synergistic apoptosis-inducing effects were significantly decreased not only by NAC (2.51% or 7.70% of apoptotic cells) but also by BHT (12.79% of apoptotic cells) and *α*-tocopherol (8.55% of apoptotic cells) in HL-60 and Jurkat cells, respectively. These results together suggest that the ATO/CMEMM-induced apoptosis in leukemia cells was mediated by oxidative stress.

### 3.4. ATO/CMEMM Combination Induces Apoptosis via the Intrinsic Pathway

**Figure 6:**
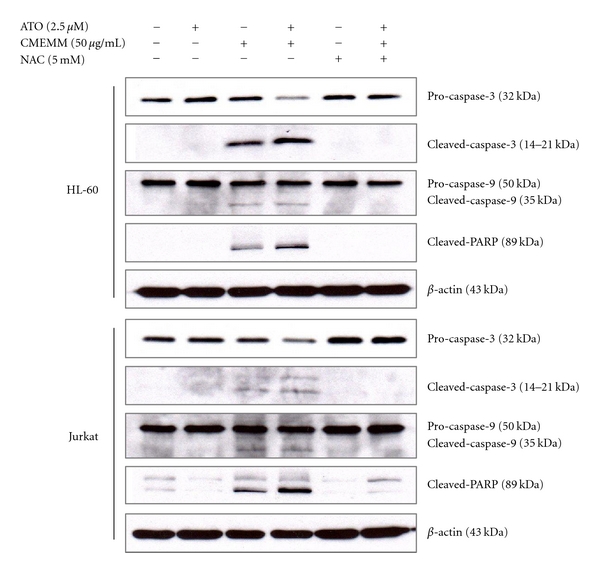
Expressions of apoptosis-related proteins in leukemia cells treated with arsenic trioxide (ATO), crude methanolic extract of *Mucuna macrocarpa* (CMEMM), and/or *N*-acetyl cysteine (NAC). Whole cell lysates were prepared from HL-60 or Jurkat cells treated with 2.5 *μ*M ATO, 50 *μ*g/mL CMEMM, 5 mM NAC, or indicated combinations for 24 h. Proteins as indicated were analyzed by Western blotting with *β*-actin as loading control. Representative blots shown were from one of three independent experiments.

Induction of apoptosis through the intrinsic, mitochondrial pathway was further investigated in leukemia cells via assessment of cleavage/activation of caspase-3, caspase-9, and PARP. As shown in Figure 6, higher levels of cleaved caspase-3, caspase-9, and PARP were present in HL-60 and Jurkat cells exposed to the combination of ATO/CMEMM than with ATO alone. Additionally, addition of NAC in the combination treatment significantly inhibited the action of cleavage in these apoptosis-regulatory factors.

## 4. Discussion and Conclusions

Since ATO has had remarkable success in the treatment of APL, an increasing number of preclinical studies to broaden the therapeutic potential have been reported during the last decade. Unfortunately, ATO as a single agent has demonstrated only limited benefit in non-APL hematological malignancies. Recently, combinatorial treatment has been considered a good strategy to enhance ATO cytotoxicity by combining it with a prooxidant agent or natural components in vitro. In the present study, the results revealed that antileukemic activity of ATO was potentiated by combining it with a folk remedy, CMEMM through enhancing growth inhibition (Figure 1) and increasing the induction of apoptosis via a ROS-dependent mechanism (Figures 2, 3, 4, and 5) and intrinsic, mitochondria-mediated pathway (Figure 6). Accordingly, findings from the present study provide scientific support as well as the elucidation of the underlying mechanisms of synergistic antileukemic activities mediated by ATO and CMEMM.

Drug interaction of ATO and CMEMM was evaluated by CI values (Table 1). Interestingly, ATO and CMEMM synergistically inhibit cell growth on HL-60 cells, whereas variable effects were exerted by combining ATO with different doses of CMEMM on Jurkat and Molt-3 cells. As combined 2.5 *μ*M ATO with 75 *μ*g/mL CMEMM or 5 *μ*M ATO with 25 *μ*g/mL CMEMM, the drug interaction indicated antagonism in Jurkat or Molt-3 cells, respectively. The discrepancy in drug interaction between promyelocytic and lymphoid leukemia cells could be attributed to differences in cell-type specific molecular or genetic factors [20]. For instance, the degradation of PML-RARalpha protein, an APL marker protein resulting from chromosomal t(15; 17) translocation, or the reduction of genomic methylation level may be involved in drug interactions of antileukemic agents in different leukemia cells [21–23]. For future therapeutic application, the determination of optimum dose of ATO/CMEMM-combined treatment will be a worthwhile task. Furthermore, the synergism or antagonism determination is entirely based on the mass-action law principle of the median-effect equation. Since the synergism is not observed in general when apoptosis is studied in Figure 2 and Figure 6, it should be noted that synergism or antagonism determination is mechanism independent, drug unit independent, and dynamic order independent [18]. Nevertheless, the results of annexin V-FITC/PI analyses showed that the synergistic effects of apoptosis were induced by combining ATO at a clinically achievable concentration (2.5 *μ*M) with 50 *μ*g/mL CMEMM in HL-60 and Jurkat cells (Figure 5). 

 ROS, such as superoxide anion and hydrogen peroxide, are toxic byproducts of cellular metabolism. Although ROS at moderate concentration are not toxic but rather act as signaling molecules, their overproduction and/or accumulation can cause nonspecific damage to proteins, nuclear acids, and other cellular components [24, 25]. ATO-stimulated ROS generation in cultured cells is well recognized. In vitro studies have indicated that the pathway responsible for ATO-induced apoptosis on leukemic cells is through NADPH oxidase [26], mitochondrial electron transport chain [27], or the inhibition of antioxidant enzymes such as thioredoxin reductase (TrxR) [28] and glutathione peroxidase (GPx) [29], thereby inducing ROS-dependent apoptosis. For these reasons, in the current study we demonstrated the important role of ROS generation in the ATO/CMEMM-mediated apoptosis (Figures 4 and 5). Similarly, recent studies reported that ATO-provoked apoptosis could be augmented by natural polyphenols, such as quercetin, genistein, and curcumin, in human leukemia cells through the generation of a prooxidant environment [14, 15, 30]. Taken together, prooxidant environment can be induced by polyphenols in combination with ATO to result in the potentiation of antileukemic effect via overproduction of ROS.

 To discern whether ROS overproduction is a late consequence of damage (e.g., mitochondrial damage) in ATO/CMEMM-treated leukemia cells, the cell proliferation assay and superoxide production measurement were also performed at an earlier stage. Our preliminary results showed that no significant growth inhibition was observed in ATO/CMEMM-treated Jurkat and HL-60 cells after 16 h of exposure (data not shown). In the meanwhile, the superoxide productions of ATO/CMEMM-treated Jurkat cells increased in a time-dependant manner within 4 to 16 h period while that of ATO/CMEMM-treated HL-60 cells slightly increased at 16 h (Figure 4). Therefore, before the cytotoxicity was evident the increase of ROS accumulation in Jurkat cells was more obviously than HL-60 cells. Furthermore, we also performed mitochondrial superoxide production measurement in ATO/CMEMM-treated leukemia cells. The results showed that mitochondrial superoxide production significantly increased in ATO/CMEMM-treated Jurkat and HL-60 cells after 24 h of exposure, and the increasing pattern was similar to that of intracellular superoxide production (data not shown). As we know, oxidative stress elicited by overproduction of ROS in mitochondria may trigger apoptosis by inhibition of the ubiquitination and proteasomal degradation system or activation of protein kinase C-*δ* [31]. These preliminary results implied that oxidative stress induced by mitochondrial superoxide production may play an important role in ATO/CMEMM-induced apoptosis. 

Moreover, changes in the intracellular milieu of the cells, such as alterations in the redox environment, have been indicated as important regulators of the progression to apoptosis [32]. Depletion of glutathione (GSH) or superoxide dismutase (SOD) has been shown to play an important role in cell survival and act as a target for the selective killing of cancer cells [33, 34]. However, our preliminary results showed that relative levels of total intracellular GSH or SOD did not significantly decrease in ATO/CMEMM-treated HL-60 or Jurkat cells as compared to controls (data not shown). Since the exact mechanisms involved in the regulation of apoptosis by GSH or SOD remain elusive, other regulators of apoptosis such as glutathionylation (protein-SSG) and nitrosylation (protein-SNO) may be involved in redox-directed cytotoxicity [35]. Therefore, further investigation may focus on the role of oxidative protein modifications in the redox environment generated by combinatorial treatment of ATO and CMEMM.

To determine whether ATO/CMEMM combination induces caspase-dependent cell death, we further evaluated ATO/CMEMM-induced apoptosis in the presence of a pan-caspase inhibitor, z-VAD-fmk by flow cytometry. After 24 h of combination treatment of 2.5 *μ*M ATO and 50 *μ*g/mL CMEMM, the frequency of apoptosis was greatly reduced by the addition of 25 *μ*M z-VAD-fmk in Jurkat cells but not in HL-60 cells (data not shown). It is noteworthy that the percentages of necrotic cells, annexin V-positive, and PI-positive, in ATO/CMEMM-treated HL-60 and Jurkat cells, were 13.17% and 2.22%, respectively (Figure 5). This implied that z-VAD-fmk did not reduce apoptosis generation in HL-60 cells which may result from the distinct mode of necrotic death. Also, the possibility of other caspase-independent modes of death may be considered.

 Dried stems of *M. macrocarpa* are used as blood-activating and stasis-resolving herbs to relieve symptoms related to leukemia in folk medicine [10]. Phytochemical components of CMEMM were analyzed by chromatography and spectroscopy in our previous study [11]. Several bioactive isoflavones including calycosin, afrormosin, and genistein were identified in CMEMM with pure markers standards using HPLC. Calycosin and afrormosin have been reported to exhibit growth inhibition in U937 lymphoma cells [36] and inhibitory effects on TPA-induced skin tumor promotion in mouse [37], respectively. Moreover, genistein has been found to be an antileukemic agent [38] and also a prooxidant to potentiate ATO-provoked apoptosis in human leukemia cells via reactive oxygen species generation [15]. The concentration of genistein to enhance ATO-provoked apoptosis was at 50 *μ*M after 24 h of exposure in HL-60, THP-1, Jurkat, RPMI-8866, and U937 human leukemia cells. Since our previous HPLC quantitative analysis showed that the content of genistein was 0.001% in dried stems of *M. macrocarpa *(data not shown), it was reasonable to assume that the low content of genistein was not the main component in CMEMM that enhanced ATO cytotoxicity in HL-60 and Jurkat cells. This implied that the synergistic antileukemic activity of cotreatment of ATO and CMEMM may have resulted from the interaction of multiple flavonoids instead of a single effective constituent such as genistein. Furthermore, our previous mouse xenograft study showed that no obvious acute toxicity of CMEMM at 500 mg/kg/day was observed during the treatment period [11]. The novel finding is that ATO can be combined with clinical achievable doses of CMEMM in inhibiting growth of leukemia cells.

In conclusion, we presented evidence that treating human leukemia cells with CMEMM led to the potentiation of ATO cytotoxicity via a ROS-dependent mechanism. The synergistic drug interaction correlates with the prooxidant action of CMEMM, as measured by ROS overproduction, and the activation of apoptosis-regulatory proteins, caspase-3 and caspase-9. Consequently, our results suggest that combinatorial treatment of ATO and CMEMM may provide a beneficial therapeutic approach in the future.

## Figures and Tables

**Table 1:** CI values for the combination of ATO and CMEMM in leukemia cell lines after 24 h of treatment.

Drug combination		Cell line					
ATO	CMEMM	HL-60		Jurkat	
(*μ*M)	(*μ*g/mL)	CI	Description	CI	Description	CI	Description
2.5	25	0.644	Synergism	1.043	Nearly additive	0.772	Moderate synergism
2.5	50	0.683	Synergism	0.802	Moderate synergism	0.935	Nearly additive
2.5	75	0.888	Slight synergism	0.820	Moderate synergism	1.155	Slight antagonism
5	25	0.887	Slight synergism	1.446	Moderate antagonism	0.852	Slight synergism
5	50	0.610	Synergism	0.868	Slight synergism	0.850	Moderate synergism
5	75	0.843	Moderate synergism	0.899	Slight synergism	1.021	Nearly additive

CI: combination index; ATO: arsenic trioxide; CMEMM: crude methanolic extract of *Mucuna macrocarpa.*
